# Transition Toward Multimodal Integration in Cancer Therapy: Biological Rationales, Clinical Efficacy, and Pharmacological Challenges

**DOI:** 10.7759/cureus.115561

**Published:** 2026-08-31

**Authors:** Khwairakpam Devendra Singh

**Affiliations:** 1 Department of General Surgery, Shija Academy of Health Sciences, Imphal, IND

**Keywords:** artificial intelligence, cancer therapy, immunotherapy, multimodal treatment, precision oncology

## Abstract

Cancer treatment is increasingly moving beyond single-agent strategies because tumor heterogeneity, clonal evolution, immune escape, and microenvironmental protection frequently limit durable disease control. Existing evidence remains fragmented across cancer types, treatment combinations, sequencing approaches, biomarker systems, and long-term safety outcomes, creating uncertainty regarding which patients benefit most from integrated therapy. This narrative review examines the biological rationale, clinical applications, treatment sequencing, precision tools, toxicity considerations, and implementation challenges associated with multimodal oncology. Relevant literature on surgery, radiotherapy, chemotherapy, targeted therapy, immunotherapy, cellular therapy, locoregional interventions, liquid biopsy, and artificial intelligence was synthesized thematically. The available evidence indicates that coordinated treatment can improve local and systemic control, delay resistance, enhance immune activation, and support organ-preserving or adaptive strategies in selected malignancies. Benefits are greatest when combinations are mechanistically justified and guided by tumor biology, disease stage, and patient fitness. Major barriers include overlapping toxicity, variable biomarker performance, limited comparative data, financial burden, and unequal access to advanced diagnostics and therapies. Multimodal care should not be equated with indiscriminate treatment intensification. Its future depends on validated predictive markers, optimized sequencing, real-time response monitoring, adaptive trials, and multidisciplinary decision-making. Precision-guided integration is likely to define the next phase of personalized cancer care.

## Introduction and background

Cancer treatment has shifted from an anatomically focused approach to a molecularly informed and immune-based approach [1]. While surgery, radiotherapy, and cytotoxic chemotherapy continue to be the core of oncology, targeted agents, immunotherapies (immune checkpoint inhibitors), antibody-drug conjugates, cellular therapies, and radioligands have broadened therapeutic options [1]. Even with these progressions, it is challenging to obtain long-term disease control in many solid and hematological malignancies. Cancer is not a single homogeneous mass but rather a complex environment of genetically, epigenetically, metabolically, and phenotypically distinct subpopulations. This variation is between patients, amongst metastases, and even within single metastases, resulting in variable sensitivity to therapeutic intervention and the presence of therapeutically resistant cell clones. Tumor heterogeneity refers to these biological differences within a single tumor, between metastatic sites, or among patients, which can result in variable treatment sensitivity and the persistence of resistant cell populations [1].

The use of monotherapy remains clinically valuable because it enables clearer evaluation of therapeutic efficacy, toxicity, and treatment delivery. However, a single drug or modality may target only one component of a complex disease process [2]. Resistant cells can survive and proliferate while sensitive populations are eliminated. Clonal evolution describes the selection and expansion of tumor-cell populations with survival advantages during disease progression or treatment. Epigenetic reprogramming refers to treatment-associated changes in gene regulation that occur without alteration of the underlying DNA sequence, whereas lineage plasticity describes the ability of malignant cells to shift their cellular phenotype or differentiation state. Additional resistance mechanisms include secondary mutations, pathway reactivation, altered drug transport, enhanced DNA repair, and metabolic adaptation [2]. The tumor microenvironment provides another layer of protection through hypoxia, abnormal vasculature, extracellular-matrix remodeling, and stromal signaling, which refers to molecular communication between tumor cells and surrounding fibroblasts, immune cells, endothelial cells, and other non-malignant components [3].

These mechanisms can be understood clinically as an evolving therapeutic system in which treatment suppresses sensitive disease while creating selective pressure that may permit resistant populations to emerge. Multimodal treatment therefore aims to address complementary disease compartments and resistance mechanisms rather than simply increasing treatment intensity. Its clinical value should be judged through outcomes such as overall survival, progression-free survival, treatment response, disease control, and toxicity, rather than inferred solely from biological plausibility. Multimodal treatment involves the use of two or more therapeutic methods for complementary local and systemic disease control [4]. Surgery may remove macroscopic disease, radiotherapy may control locoregional microscopic deposits, and systemic treatment may target disseminated or occult malignant cells [4]. Modern integration increasingly uses mechanism-based approaches intended to inhibit parallel or compensatory pathways, enhance tumor-antigen release, normalize tumor vasculature, reverse immune suppression, or increase susceptibility to cell death [5]. Accordingly, multimodal therapy should not be interpreted as a universal replacement for monotherapy; single-agent treatment remains appropriate in selected cancers and clinical settings, whereas combination strategies should be individualized according to tumor biology, disease stage, expected benefit, toxicity, and patient fitness.

This integrated approach is increasingly supported by emerging clinical evidence. Combination chemotherapy can be made more effective by inducing immunogenic cell death and depletion of suppressive populations of immune cells, which will enhance immune recognition [6]. There are multiple reasons for using radiation in combination with immune checkpoint blockade: radiotherapy can modify antigen presentation, inflammatory signaling, and the recruitment of local immune cells [7]. Dominant oncogenic drivers can be targeted, together with escape pathways that contribute to treatment resistance [8]. Pathway-specific inhibitors have offered disease control benefits to endocrine therapy in hormone-dependent malignancies and targeted therapy with immunotherapy or antiangiogenic drugs in certain malignant diseases in advanced stages [9]. Other types of cell therapy, bispecific antibodies, cancer vaccines, oncolytic viruses, and gene-based treatments are also being studied as elements of multidimensional therapy programs, not as standalone treatments [10].

To maximize the benefit of the multimodal approach to oncology, proper selection, timing, dose, sequence, and duration of therapy are crucial. Just higher intensity does not mean better outcomes [10]. Combining medications that are not well indicated can add to myelosuppression, organ toxicity, immune-related adverse events, perioperative complications, financial cost, and impact on quality of life. To find the patients best suited to benefit and those that can be treated with de-escalation, there is a need for predictive and pharmacodynamic markers [11]. Genomic profiling, transcriptomics, spatial analysis, single-cell technologies, circulating tumor DNA, and functional drug testing can provide support in the classification of the disease, detection of minimal residual disease, and monitoring of treatment responses as they happen. AI could further combine clinical, pathological, imaging, and molecular info to forecast treatment responses, toxicity, and best therapeutic sequence [12].

This approach is being adopted on multiple levels, including multidisciplinary tumor boards and adaptive clinical-trial platforms. Precision-guided integration represents a shift from static cancer treatment toward dynamic management across the treatment trajectory [12]. Multimodal therapy also introduces important pharmacokinetic (PK) and pharmacodynamic (PD) complexity. Drug exposure, metabolism, clearance, dose-response relationships, target engagement, treatment sequence, and cumulative toxicity can influence the overall effect of a combination. Polytherapy therefore cannot be viewed as a simple addition of individual treatments, because interactions may be additive, synergistic, antagonistic, or toxicity-limited, making regimen design both mathematically and clinically complex. Although monotherapy remains appropriate in selected cancers and clinical settings, multimodal treatment requires careful identification of patients most likely to benefit to address heterogeneity, resistance, recurrence, and metastatic progression. At present, evidence remains scattered across cancer types, treatment combinations, biomarker systems, and therapeutic sequences. Limited comparative evidence is available to determine which patients benefit most, which combinations provide clinically meaningful synergy, and how timing, duration, toxicity, and cost can be optimized in routine practice.

Objective of the review

This narrative review aims to evaluate the biological and clinical rationale for multimodal cancer therapy and to assess its reported effects on clinically relevant outcomes, including overall survival (OS), progression-free survival (PFS), disease control, treatment response, recurrence, and treatment-related toxicity where these outcomes are available in the cited literature. It further examines overlapping toxicity profiles, treatment sequencing, biomarker-guided patient selection, and the balance between therapeutic benefit and cumulative toxicity across combinations of surgery, radiotherapy, chemotherapy, targeted therapy, immunotherapy, and emerging technologies. Because this is a narrative review rather than a quantitative meta-analysis, statistical significance is interpreted as reported in the individual studies rather than recalculated or pooled across heterogeneous treatment settings.

Methodology

The narrative review approach was used to investigate the adoption of a transition from monotherapy to multimodal integration in cancer treatment. PubMed, Scopus, Web of Science, and Embase databases were used to retrieve relevant literature. The search included all the terms associated with cancer treatment, combination therapy, multimodal oncology, chemotherapy, radiotherapy, surgery, immunotherapy, targeted therapy, cellular therapy, precision oncology, treatment resistance, liquid biopsy, biomarkers, and artificial intelligence. The publications included in this review were those published between January 2018 and July 2026 that had undergone peer review. Original clinical investigations, randomized and nonrandomized trials, large case-control or cohort studies, translational research, systematic reviews, meta-analyses, consensus statements, and authoritative guidelines on integrated cancer management were eligible sources. Only articles that discussed either the biological rationale, therapeutic sequencing, clinical effectiveness, safety, predictive markers, or implementation challenges for combining two or more therapeutic modalities were selected. Articles that did not address combined care, were isolated case reports, conference abstracts, were editorials, preclinical studies without clinical interpretation, non-peer-reviewed articles, duplicate articles, or were not available in English were excluded. Evidence selected was arranged thematically based on the mechanisms of resistance, major therapeutic combinations, timing of treatment, precision-guided selection, toxicity, and future clinical translation. As this was a narrative review, no formal Population, Intervention, Comparison, Outcome (PICO) framework, quantitative statistical synthesis, meta-analysis, meta-regression, pooled effect estimates, P-values, or confidence intervals were generated. The evidence was synthesized qualitatively and thematically because of heterogeneity in cancer types, treatment modalities, study designs, and reported outcomes; therefore, additional statistical review was not required.

## Review

Tumor heterogeneity and biological rationale for multimodal integration

The process of cancer is characterized by a series of genetic, epigenetic, and microenvironmental alterations that lead to significant diversity within and between tumors [13]. Multiple subpopulations of cells with distinct mutations, transcriptomes, metabolomes, and treatment susceptibilities can be present within a single malignancy. Intratumoral heterogeneity significantly impacts disease progression, metastatic potential, and response to therapy, as each cellular clone responds differently to the same therapy [13]. The make-up of a tumour is also not static; as the treatment progresses, selective pressures may shift the proportions of sensitive and resistant populations within the tumour [14]. Clonal evolution is a process in which malignant cells acquire new molecular changes that confer a survival or proliferative advantage. Tumors can change their behavior over time (termed secondary mutations); amplified oncogenic drivers, alterations in DNA repair pathways, and activation of alternative signaling networks can reshape tumor behavior [14]. Phenotypic plasticity also allows cancer cells to shift among epithelial, mesenchymal, stem-like, and lineage-altered states without permanent genetic alterations [15]. These changes can affect the invasion and metastatic dissemination of the cancer, immune recognition, and immune response to treatment.

The tumour microenvironment plays a role in this changing bio-system. Malignant cells interact with cancer-associated fibroblasts, endothelial cells, immune cells, extracellular-matrix elements, and hypoxic areas via biochemical and mechanical signals [16]. These interactions can lead to angiogenesis, suppression of the immune system, invasion of tissue, and adaptation to therapeutic stress. Primary tumors can also develop molecular differences when they grow and spread to secondary locations, leading to spatial variation of the disease at different locations [16]. Cancer can be viewed as an ecosystem, and this is the conceptual foundation of integrated treatment. From a pharmacological perspective, interactions between anticancer agents should be classified as synergistic, additive, or antagonistic rather than assumed to be beneficial solely because the agents have different mechanisms of action. Established approaches such as the combination index (CI) and isobologram analysis provide quantitative frameworks for evaluating these interactions. A CI <1 indicates synergism, CI = 1 indicates an additive effect, and CI > 1 indicates antagonism, while isobolographic analysis compares observed combination effects with the expected line of additivity [17]. These approaches help distinguish true pharmacological synergy from simple additivity or antagonism and support more rational selection of multimodal regimens. This view is in favor of choosing a therapy that is personalized to the patient because of the composition of the tumor, the stage of the disease, the molecular characteristics of the tumor, and its evolution over time.

The rationale behind multimodal cancer therapy is that diseases of biologic complexity are not likely to be controlled by targeting a single pathway, cellular population, or anatomical compartment of the disease [18]. Multiple modalities are effective because tumors are heterogeneous, with exposure of the tumor cells to different mechanisms of injury. Local control is achieved by surgery and radiotherapy, while systemic therapies target circulating tumour cells, micrometastatic deposits and distant tumours. When integrated, they can decrease the likelihood of residual disease persisting by one resistance mechanism [18]. Combination strategies may involve vertical or horizontal pathway inhibition. Inhibition of vertical targets several parts of the same oncogenic pathway, lessening the chances of re-activation downstream. Horizontal inhibition also inhibits parallel pathways that can substitute for each other if one target is inhibited, thereby removing parallel pathways [19]. These strategies are especially applicable to tumors that are regulated by cross-talking signaling pathways. Temporal vulnerability can also be used with multimodal treatment. Cytotoxic therapy could shrink the tumor before surgery, radiotherapy can induce antigen release, and antiangiogenic therapy may temporarily normalize the tumor vasculature, potentially enhancing the infiltration of immune cells or delivery of therapeutic agents [20]. Synergy will be realized when the therapeutic effect of the combination is greater than the sum of the parts. The mechanisms by which chemotherapy and radiation could improve response to immune checkpoint blockers (ICBs) include increased immunogenic cell death, enhanced antigen presentation, and alteration of the tumor milieu [21]. Targeted agents can also overcome immune exclusion and/or inhibit molecular pathways involved in resistance to immunotherapy. The treatment interaction must be understood to be rational, i.e., by a mechanistic approach. Combinations that are chosen due to an independent single agent activity can yield overlapping toxicity without enhancing efficacy. Optimally designed multimodal therapy should combine biological complementarity, the correct dose, sequence, and patient selection [22]. This framework helps differentiate precision-guided integration from indiscriminate intensification of treatments and helps guide the development of regimens based on tumor biology and not just on treatment availability. The main reasons for mono failure and the suggested multimodal treatments are recapped in Table 1.

**Table 1:** Major Causes of Monotherapy Failure

Cause	Effect	Multimodal Solution	Reference
Tumor heterogeneity	Variable treatment response	Combine therapies with different mechanisms	[1,13]
Acquired resistance	Disease progression after initial response	Dual or multiple pathway inhibition	[2,14]
Tumor microenvironment	Reduced drug delivery and immune activity	Add immunotherapy or antiangiogenic therapy	[16,20]
Residual disease	Increased risk of recurrence	Add adjuvant systemic therapy or radiotherapy	[18]
Dose-limiting toxicity	Restricts effective monotherapy dosing	Use complementary lower-dose combinations	[18,22]

Integration of surgery, chemotherapy, and radiotherapy

Multimodal oncology is based on surgery, chemotherapy, and radiotherapy, and these are the pillars of many localized and regionally advanced cancer treatments. Surgery offers histopathological diagnosis and accurate staging of disease, cytoreduction, and control of resectable disease. It has some limitations due to microscopic residual disease, occult nodal disease, and occult distant micrometastases that may not be removed during the procedure. Perioperative chemotherapy and radiotherapy are used to decrease recurrence and enhance local or systemic disease control. Contemporary perioperative strategies increasingly incorporate immunotherapy alongside these established modalities. In the KEYNOTE-671 trial, perioperative pembrolizumab combined with neoadjuvant platinum-based chemotherapy and followed by adjuvant pembrolizumab improved outcomes in patients with resectable early-stage non-small-cell lung cancer, supporting the integration of immunotherapy with chemotherapy and surgery across the perioperative treatment pathway [23].

Neoadjuvant therapy may help reduce the size of the tumor, improve resectability, and allow for organ-sparing surgery. It also offers a radiological and pathological evaluation of the sensitivity of the treatment. Pathological response can provide prognostic data and inform postoperative management, with major or complete responses [24]. Adjuvant treatment is used to kill tumour cells after the main treatment, to lower the chances of recurrence. It is effective depending on baseline recurrence risk, tumour biology, surgical margins, nodal status and presence of predictive biomarkers [24]. Concurrent chemoradiotherapy (CCRT) is a treatment modality that is the combination of the local cytotoxic activity of radiation and the radiosensitizing and systemic activity of chemotherapy. This approach has been defined in multiple malignancies of the head and neck, thoracic, gastrointestinal, gynecological, and central nervous systems [25]. Toxicity associated with treatment can be significant, necessitating careful patient selection, supportive care, and coordination of treatment from different specialties.

Total neoadjuvant therapy has facilitated the merging of systemic therapy and radiation before surgery, especially in selected cases of locally advanced cancer. It may provide the benefit of early treatment of micrometastatic disease, benefit to finish the planned treatment, and enhance tumor regression rates [26]. Conversion therapy can also facilitate the resection of those tumors which were initially unresectable following systemic or local regional therapy. Optimized integration involves decisions about the order of treatments, when to perform surgery, the radiation fields, and recovery periods. Multidisciplinary tumor boards are still a key tool in ensuring that the oncological benefit, procedural feasibility, toxicity, and preservation of function are balanced [27]. Multimodal treatment pathways and multidisciplinary planning of localized and regionally advanced cancer are described in Figure 1.

**Figure 1:**
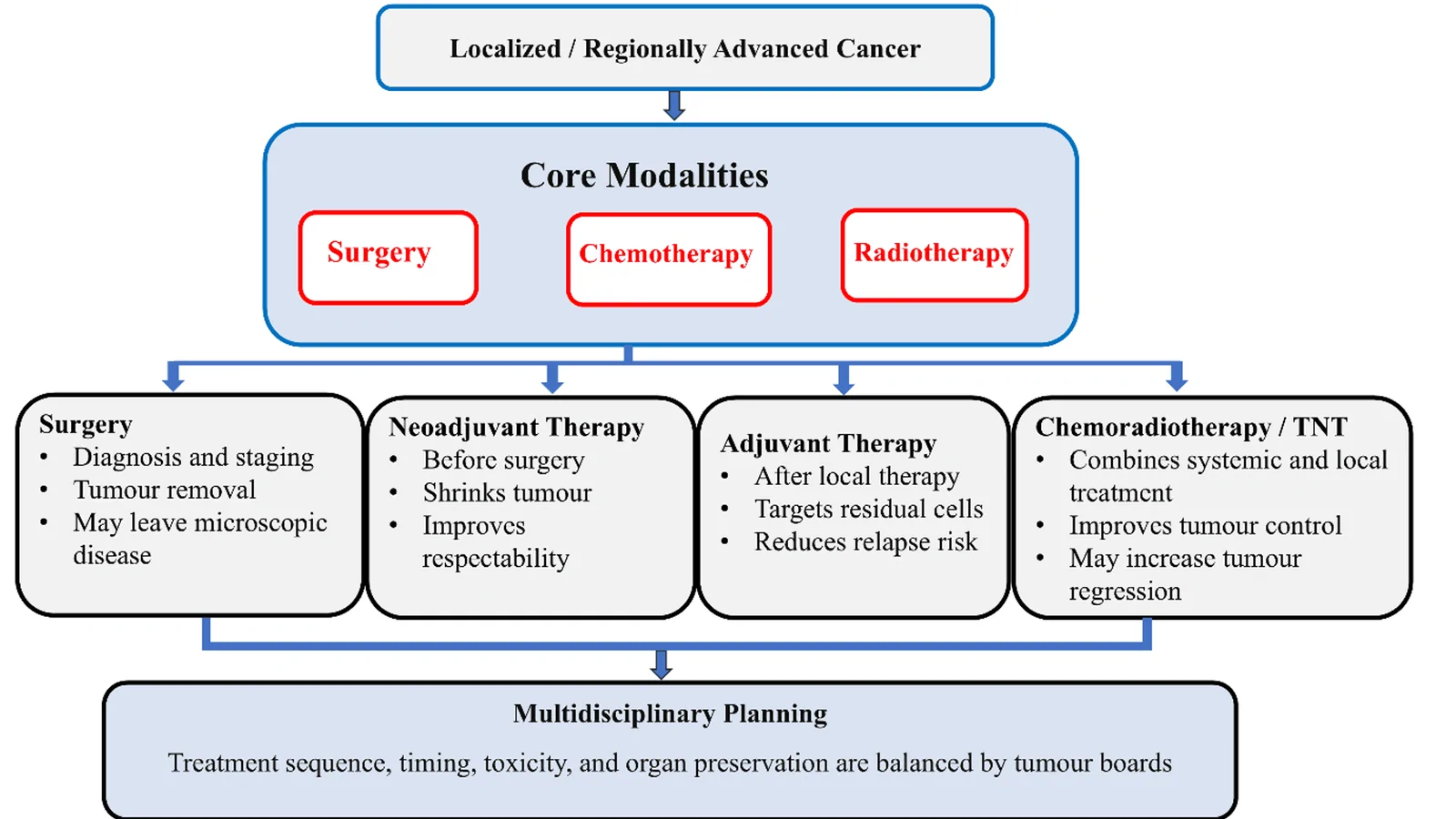
Integration of Surgery, Chemotherapy, and Radiotherapy Created by the author using Microsoft PowerPoint (Microsoft Corporation, Redmond, USA).

Immunotherapy-based multimodal strategies

While immune checkpoint inhibitors have revolutionized the care of many cancers, a small percentage of patients do not respond to immunotherapy and will not have long-term benefit [28]. The reasons for primary resistance include antigenicity that is too weak, poor antigen presentation, exclusion of effector lymphocytes, immunosuppressive signaling, and lack of activation of immune cells [28]. Acquired resistance can develop in several ways, including reduced expression of antigens, impairment of interferon signaling, or adaptive up-regulation of alternate inhibitory pathways [28]. The rationale for combining immunotherapy with other modalities is to try to overcome these barriers. Chemotherapy can release tumor antigens, induce immunogenic cell death, and decrease populations of immune cells that suppress the immune system. Antibody-drug conjugates are also increasingly being integrated with immune checkpoint inhibition. In the EV-302 trial, enfortumab vedotin combined with pembrolizumab demonstrated substantial clinical benefit as first-line therapy for previously untreated locally advanced or metastatic urothelial carcinoma, providing contemporary evidence for integrating targeted cytotoxic delivery with immunotherapy [29]. Radiotherapy can also boost antigen presentation, boost inflammatory signaling, and change the local immune context in such a way. It is being tested in combination with immune checkpoint blockades in localised, oligometastatic and metastatic settings; the best radiation dose, sequence and fractionation have yet to be determined [30].

Dual checkpoint inhibition involves targeting non-redundant inhibitory pathways and can enhance the breadth and persistence of immune responses [31]. This enhanced antitumor activity is accompanied by a greater burden of immune-related adverse events (irAEs) than is generally observed with single-agent checkpoint inhibition. Clinically important toxicities may involve the gastrointestinal, hepatic, endocrine, pulmonary, dermatologic, and other organ systems, and severe events can necessitate treatment interruption, systemic immunosuppression, or permanent discontinuation. Dual checkpoint blockade therefore modifies the therapeutic index by potentially improving antitumor efficacy while simultaneously narrowing the safety margin through increased and potentially overlapping immune-mediated toxicity. Careful patient selection, baseline assessment, early recognition of irAEs, and multidisciplinary toxicity management are consequently important when combination immunotherapy is considered. Another approach involving immunotherapy and antiangiogenic agents is important. Inhibition of vascular endothelial growth factor (VEGF) can normalize abnormal vasculature, decrease immune suppression, and increase infiltration by T cells [31]. New combinations are being developed, such as checkpoint inhibitors with cancer vaccines, oncolytic viruses, bispecific antibodies, cellular therapies, and epigenetic modulators. The strategies aim to render cold tumors into an inflamed and drug-responsive environment [32]. Biomarker limitations, heterogeneous responses, and overlapping toxicity still limit clinical translation. A single marker cannot be used for all tumour types to select patients. A combination of programmed death-ligand 1 expression, genomic characteristics, immune-cell composition, and disease burden may serve as a more reliable criterion for the selection of treatment [32].

Targeted therapy and precision combination approaches

Targeted therapies block molecular abnormalities that are critical for tumor growth, survival, or dissemination. They can be very effective in biomarker-selected populations, but may be less effective because of inherent heterogeneity and the fast activation of resistance pathways [33]. Precision combination therapy tries to inhibit the dominant oncogenic driver, as well as the molecular mechanisms that allow tumour escape [33]. The ability to accurately characterize the genomes and understanding of pathway interdependence is necessary for this approach. In vertical pathway blockade, several agents are used to block various parts of the same pathway. When both an upstream driver and a downstream effector are inhibited, there may be more pathway suppression and a delay in reactivation [34]. Horizontal blockade refers to pathways that are parallel with the main pathway and will sustain a pathway even in the presence of an inhibited pathway. This approach may be applicable in signaling networks that are functionally redundant, but could make them more toxic and decrease the therapeutic window if simultaneous inhibition is used.

A few clinically proven combinations are examples of this [35]. Human epidermal growth factor receptor-2-targeted agents can be used in combination with chemotherapy, endocrine therapy, and an antibody-drug conjugate. In some mutation-driven cancers, inhibiting the pathways decreases the reactivation of the pathways. Tumors that have impaired DNA repair can be treated with poly(adenosine diphosphate-ribose) polymerase inhibitors along with antiangiogenic, immune, or DNA-damaging drugs [35]. In hormone receptor-positive disease, cyclin-dependent kinase 4/6 inhibitors used in combination with endocrine therapy slow down the progression of the cell cycle and resistance. Immunotherapy and targeted therapy can also be combined with antiangiogenic agents to change tumor vascularity and immune suppression [36].

Tumor evolution with time should be taken into account in precision combination therapy. Molecular profiling done at diagnosis may not reflect the biology of recurrent/metastatic disease. In cellular immunotherapy, treatment efficacy may be influenced by target-antigen expression, persistence and functional fitness of infused T cells, and tumor-associated mechanisms that permit immune escape, emphasizing the need for treatment strategies that account for evolving tumor biology [37]. For this to be realized, it is necessary to use validated biomarkers, standardized assays, appropriate toxicological monitoring, and prospective evidence that the chosen combination does more than improve on a molecular level; it actually enhances clinically meaningful outcomes. Pathway blockade, combinations of treatments, and molecular monitoring are summarized in Figure 2.

**Figure 2:**
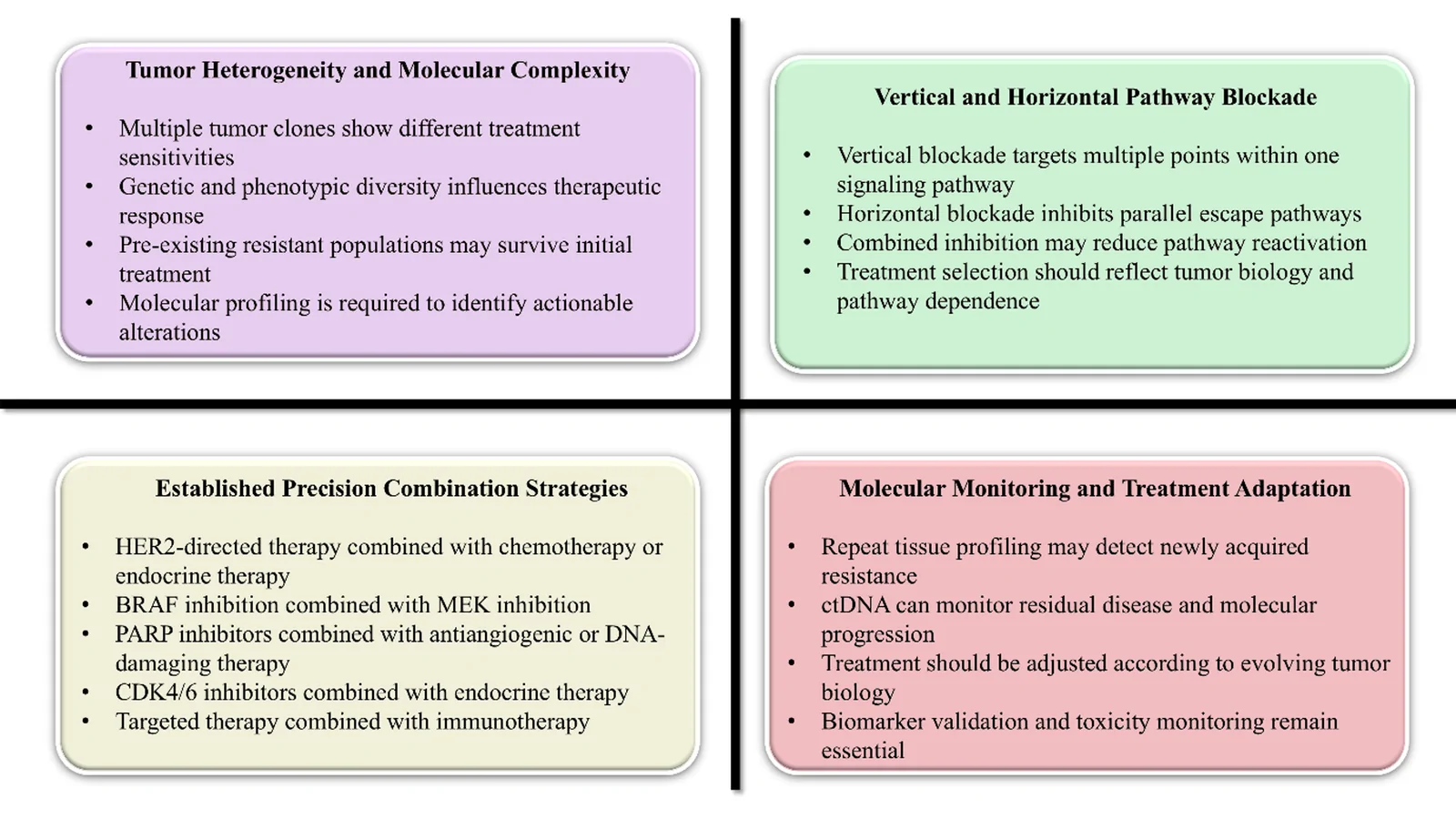
Precision Combination Strategies in Cancer Therapy Created by the author using Microsoft PowerPoint (Microsoft Corporation, Redmond, USA). BRAF: B-Raf kinase; CDK: cyclin-dependent kinase; HER2: human epidermal growth factor receptor 2; MEK: mitogen-activated protein kinase kinase; PARP: poly(ADP-ribose) polymerase; ctDNA: circulating tumor DNA.

Cellular, gene-based, and locoregional therapies

Cellular therapies have introduced new mechanisms for recognizing and eliminating malignant cells. Tumor-infiltrating lymphocyte (TIL) therapy has demonstrated clinically meaningful antitumor activity in advanced melanoma and represents an established form of adoptive cellular immunotherapy in selected patients [38]. Engineered T-cell receptor (TCR) approaches may further broaden the range of tumor-associated intracellular and surface antigens that can be targeted. Checkpoint inhibitors, cytokines, vaccines, targeted agents, and lymphodepleting regimens are increasingly being investigated in conjunction with cellular therapies to improve immune-cell expansion, persistence, and antitumor activity.

Multimodal therapy also incorporates locoregional interventions. Thermal ablation, cryoablation, embolization, regional chemotherapy, and other image-guided approaches can provide focused tumor control with limited systemic exposure and may be integrated with surgery, systemic therapy, or immunotherapy in selected malignancies [39]. Local destruction of the tumor may result in the release of antigens and inflammatory mediators, providing a biological rationale for combination with immune-based treatments.

Chimeric antigen receptor T (CAR T) cell therapy has achieved substantial activity in selected hematological malignancies, but its application to solid tumors remains challenging because of heterogeneous antigen expression, limited trafficking and infiltration, stromal barriers, an immunosuppressive tumor microenvironment, T-cell exhaustion, and treatment-associated toxicities [40,41]. Strategies under investigation include dual-target receptors, armored CAR-T cells, local delivery, and genetic engineering approaches designed to enhance target recognition, cellular persistence, and resistance to inhibitory signals [40,41]. Unlike conventional pharmacological agents, CAR-T cells can undergo in vivo expansion and persistence after infusion; consequently, cellular kinetics and treatment-related toxicities such as cytokine release syndrome and neurotoxicity require careful longitudinal monitoring.

Radiotherapy may also complement immune-based treatment by promoting tumor-antigen release, modifying inflammatory signaling, and enhancing immune-cell recruitment within the tumor microenvironment [42]. The future clinical value of cellular and multimodal approaches will depend on scalable manufacturing, manageable toxicity, sustained therapeutic activity, appropriate patient selection, and demonstration of clinically meaningful benefit. Table 2 shows the principal multimodal combination treatments and purposes of use.

**Table 2:** Common Multimodal Cancer Treatment Strategies

Treatment Combination	Main Purpose	Reference
Surgery plus chemotherapy	Control local and microscopic systemic disease	[23,24]
Chemotherapy plus radiotherapy	Improve local control and radiosensitivity	[25,30]
Chemotherapy plus immunotherapy	Enhance tumor killing and immune activation	[5,21]
Radiotherapy plus immunotherapy	Increase antigen release and immune response	[7,30]
Dual targeted therapy	Block dominant and escape pathways	[4,32]
Cellular therapy plus immunotherapy	Improve immune-cell activity and persistence	[10,41]

Treatment sequencing, timing, and adaptive therapy

The effectiveness of the multimodal approach to cancer treatment relies on the combination of treatments used as well as the timing and sequence of their delivery [43]. Concurrent treatment can have the greatest biological interactions but may also increase acute toxicity. Sequential therapy can enhance tolerability and allow recovery between treatment modalities, but may decrease synergy or systemic disease control [43]. The best approach will depend on the type of tumor, stage of disease, nature of treatment, and patient's fitness. Neoadjuvant therapy is used before definitive local therapy to reduce tumour burden, to increase the likelihood of local therapy being resectable and to test the biological response to treatment. The purpose of adjuvant therapy is to treat any remaining microscopic disease following surgery or radiotherapy [44]. Maintenance therapy should be a less intensive treatment to help maintain the disease in control, while consolidation therapy will be used to further improve the initial response. A clear therapeutic goal and specifically established criteria for continuation, modification, and discontinuation of each phase are required [44].

Adaptive therapy breaks from the maximization of the dose until the disease progresses. It adjusts therapy dynamically by responding to patterns of responses, tumour burden, biomarkers, and new patterns of resistance signals [45]. Reducing dose, giving treatment breaks, or dose-selective continuation can maintain sensitive cells that inhibit competitive expansion, perhaps postponing the growth of resistant clones. The decision of response-guided escalation versus de-escalation could be useful in patients with inadequate early control and in patients with deep molecular or pathological response, respectively, to minimize unnecessary toxicity [45]. New technologies such as circulating tumor DNA (ctDNA), functional imaging, and serial molecular profiling may allow for earlier detection of residual disease and resistance that is not detectable by conventional radiological assessment [46]. These tools may help switch treatment before clinically manifest disease. However, their use is still restricted due to the variability in the assays, the lack of clarity around the thresholds, and the lack of robust evidence that changing treatment based on biomarkers would lead to better survival [46]. Further trials should investigate concurrent versus sequential therapies, establish optimal duration of therapy, and also include adaptive decision rules. Treatment sequencing should ultimately be based on biology, not a specific sequence, used across all patients.

Biomarkers, liquid biopsy, and artificial intelligence

Biomarkers predictive of treatment benefit, monitors of response, and markers of resistance are needed to support precision-guided multimodal therapy [47]. Oncogenic driver alterations, programmed death-ligand 1 expression, microsatellite instability, tumor mutational burden, and homologous recombination deficiency are established biomarkers. Their clinical utility is different depending on the cancer type, assay, and treatment setting. A marker that has been predictive of response to one agent may not be predictive of benefit from a combination regimen [47]. Liquid biopsy is a minimally invasive approach that can be used to identify circulating tumor DNA (ctDNA), circulating tumor cells (CTCs), and other tumor-derived products. Radiological progression may not be seen until the molecular response or minimal residual disease, or the emergence of resistance has been detected by serial assessment [48]. In addition, circulating tumor DNA (ctDNA) can also carry genomic changes from various metastatic locations, minimizing some of the constraints of a single lesion tissue biopsy. Tumors with low levels of DNA shedding, little or no disease burden, and/or disease in limited areas will be less sensitive [48].

Single-cell sequencing and spatial profiling can determine the cellular composition and organization of tumor microenvironment (TME). They can detect immune-excluded regions and treatment-induced changes that cannot be discerned in bulk genome analysis, and may reveal resistant subclones [49]. Patient-derived organoids and ex vivo drug-sensitivity testing are other functional precision strategies to assess therapeutic combinations directly on the patient's tumor material [49]. AI could combine genomic, transcriptomic, pathological, radiological, and clinical information into predictive models [50]. These models may support treatment selection, toxicity estimation, response monitoring, and identification of clinically relevant patient subgroups. The algorithms need to be developed transparently, externally validated, and proper training populations need to be used, and algorithmic bias needs to be protected against [50]. Importantly, no single biomarker is likely to provide a complete representation of multimodal treatment response. Molecular, imaging, immune, and clinical variables may be combined into composite variables that would offer more accurate guidance. Their worth needs to be measured in future trials that have shown improved results, rather than simply better prediction ability. Table 3 lists some key precision tools to be used for selecting alternative treatments and monitoring those treatments.

**Table 3:** Precision Tools Used in Multimodal Oncology ctDNA: circulating tumor DNA; PD-L1: programmed death-ligand 1

Tool	Main Role	Reference
Genomic profiling	Identifies actionable mutations	[33,36]
PD-L1 testing	Supports immunotherapy selection	[9,47]
ctDNA analysis	Monitors residual disease and resistance	[36,48]
Single-cell profiling	Detects resistant cellular populations	[13,49]
Artificial intelligence	Predicts response and toxicity	[12,50]

Limitations and future directions

This review has a few limitations. The evidence available is not uniform about cancer type, stage of disease, therapy combinations, sequence of therapy, and how to assess therapeutic outcome. The studies often have very narrow patient populations, brief follow-up periods, and inadequate representation of older, frail, and socioeconomically impoverished patients. Different definitions for biomarkers, different platforms on which they were measured, and different criteria for defining a response also limit comparability and generalizability. There is limited evidence of long-term toxicity, quality of life, treatment length, and cost-effectiveness. An additional limitation is that this narrative review did not undertake formal pharmacoepidemiological data synthesis or quantitative pooling of toxicity outcomes; therefore, precise comparative toxicity thresholds across complex multimodal regimens could not be established.

The combinations should be investigated further based on mechanisms and validated predictive biomarkers. Evaluation of treatment sequencing and patient selection can be enhanced by adaptive and platform trials. Future adaptive trials should incorporate advanced biostatistical modeling to define regimen-specific toxicity thresholds, characterize cumulative and overlapping adverse effects, and identify dose, sequence, and patient-level factors associated with an unfavorable therapeutic index. The detection of circulating tumor DNA, spatial profiling, organoid models, and artificial intelligence could allow for dynamic changes to treatment. There is also a need for increased focus on toxicity prediction, treatment de-escalation, survivorship, affordability, and equity of access to treatment. The path towards multimodal oncology should be towards individualization and evidence-based treatment intensification and not indiscriminate.

## Conclusions

This review highlights the evolving role of multimodal integration in addressing tumor heterogeneity, therapeutic resistance, immune escape, and residual disease through the coordinated use of surgery, radiotherapy, chemotherapy, targeted therapy, immunotherapy, and emerging technologies. Monotherapy remains appropriate in selected localized or biomarker-sensitive cancers; however, resistance and clonal evolution can limit the durability of response in some settings. The success of multimodal treatment depends on appropriate biological selection, optimized sequencing, careful toxicity monitoring, and individualized patient assessment. Biomarkers, circulating tumor DNA, spatial profiling, functional models, and artificial intelligence may further support treatment selection and real-time adaptation. Clinical implementation should also consider survivorship, long-term toxicity, financial burden, and equitable access to treatment. Future progress will depend on mechanistically justified combinations, validated predictive tools, and adaptive clinical trials that define which patients are most likely to benefit. The future direction of oncology is therefore likely to emphasize evidence-based, precision-guided integration rather than indiscriminate treatment intensification.
